# Epigenetic pathway inhibitors represent potential drugs for treating pancreatic and bronchial neuroendocrine tumors

**DOI:** 10.1038/oncsis.2017.30

**Published:** 2017-05-15

**Authors:** K E Lines, M Stevenson, P Filippakopoulos, S Müller, H E Lockstone, B Wright, S Grozinsky-Glasberg, A B Grossman, S Knapp, D Buck, C Bountra, R V Thakker

**Affiliations:** 1 Academic Endocrine Unit, OCDEM, University of Oxford, Churchill Hospital, Headington, Oxford, UK; 2 Structural Genomics Consortium, University of Oxford, Old Road Campus, Headington, Oxford, UK; 3 Oxford Genomics Centre, Wellcome Trust Centre for Human Genetics, University of Oxford, Roosevelt Drive, Oxford, UK; 4 Neuroendocrine Tumor Unit, Endocrinology & Metabolism Service, Department of Medicine, Hadassah-Hebrew University Medical Center, Jerusalem, Israel; 5 Institute for Pharmaceutical Chemistry, Johann Wolfgang Goethe-University and Buchmann Institute for Molecular Life Sciences, Max-von-Laue-Strasse 9, Frankfurt am Main, Jerusalem, Germany

## Abstract

Cancer is associated with alterations in epigenetic mechanisms such as histone modifications and methylation of DNA, and inhibitors targeting epigenetic mechanisms represent a novel class of anti-cancer drugs. Neuroendocrine tumors (NETs) of the pancreas (PNETs) and bronchus (BNETs), which may have 5-year survivals of <50% and as low as 5%, respectively, represent targets for such drugs, as >40% of PNETs and ~35% of BNETs have mutations of the multiple endocrine neoplasia type 1 (*MEN1*) gene, which encodes menin that modifies histones by interacting with histone methyltransferases. We assessed 9 inhibitors of epigenetic pathways, for their effects on proliferation, by CellTiter Blue assay, and apoptosis, by CaspaseGlo assay, using 1 PNET and 2 BNET cell lines. Two inhibitors, referred to as (+)-JQ1 (JQ1) and PFI-1, targeting the bromo and extra terminal (BET) protein family which bind acetylated histone residues, were most effective in decreasing proliferation (by 40–85%, *P*<0.001) and increasing apoptosis (by 2–3.6 fold, *P*<0.001) in all 3 NET cell lines. The anti-proliferative effects of JQ1 and PFI-1 remained present for at least 48 hours after removal of the compound. JQ1, but not PFI-1, had cell cycle effects, assessed by propidium iodide staining and flow cytometry, resulting in increased and decreased proportions of NET cells in G1, and S and G2 phases, respectively. RNA Sequencing analysis revealed that these JQ1 effects were associated with increased histone 2B expression, and likely mediated through altered activity of bromodomain-containing (Brd) proteins. Assessment of JQ1 *in vivo*, using a pancreatic beta cell-specific conditional *Men1* knockout mouse model that develops PNETs, revealed that JQ1 significantly reduced proliferation (by ~50%, *P*<0.0005), assessed by bromodeoxyuridine incorporation, and increased apoptosis (by ~3 fold, *P*<0.0005), assessed by terminal deoxynucleotidyl transferase dUTP nick end labelling, of PNETs. Thus, our studies demonstrate that BET protein inhibitors may provide new treatments for NETs.

## Introduction

Epigenetic modifications have been reported to play critical roles in cancer development by altering the expression of tumor suppressor genes and oncogenes through mechanisms including chromatin remodelling.^1^ Chromatin consists of DNA packaged into nucleosomes, which is further organised into condensed chromosomes to act as a barrier for the transcriptional machinery, and therefore prevent gene transcription.^1^ Nucleosomes are composed of pairs of the histones (H) H2A, H2B, H3 and H4, which can be modified by enzymes that either add or remove motifs such as, acetyl, methyl, crotonyl and phosphate groups, to alter the chromatin state and therefore transcriptional activity.^1, 2^ Dysregulation of chromatin remodellers results in cancer development and progression, and thus compounds targeting chromatin-modifying proteins, represent a new class of anti-cancer drugs, as illustrated by inhibitors of histone deacetylases that have been used for treatment of lymphomas.^3^ We therefore explored the use of these compounds for the treatment of neuroendocrine tumors (NETs) of the pancreas (PNETs) and bronchus (BNETs), which are associated with a high mortality and for which current drugs have only limited efficacy. NETs are a heterogeneous group of benign and malignant neoplasms that occur in multiple different organs and have an incidence of 5.7 per 100 000 individuals per year. NETs of the gasteroenteropancreatic and bronchopulmonary tracts occur most frequently with incidences of 3 and 1 per 100 000 individuals per year, respectively.^4, 5^ Gastroenteropancreatic NETs include carcinoid tumors and PNETs, while bronchopulmonary NETs include low-grade typical carcinoids, intermediate-grade atypical carcinoids, and high-grade (>Grade 3) large cell neuroendocrine carcinomas (LCNEC) and small cell lung cancers (SCLC). PNETs may secrete hormones such as gastrin, insulin, glucagon, vasoactive intestinal polypeptide (VIP) and are referred to as gastrinomas, insulinomas, glucagonomas or VIPomas, respectively, or may be non-secreting (i.e. non-functioning). All PNETs and BNETs, which represent ~2% of all diagnosed lung tumors,^6^ can be malignant and subsequently metastasise, and the 5-year survival for patients with PNETs and high-grade (>Grade 3) BNETs (LCNECs and SCLCs) are <50% and ~5%, respectively. Current treatments for PNETs and BNETs, which include surgery, drugs (e.g. somatostatin analogues), chemotherapy (e.g. everolimus, sunitinib), radiotherapy and radionuclide therapy, are often not effective.^4, 7, 8^ PNETs and BNETs usually occur as non-familial (i.e. sporadic) isolated tumors, but can also occur as part of familial syndromes such as multiple endocrine neoplasia type 1 (MEN1). MEN1 is caused by mutations of the *MEN1* gene, which encodes the tumor suppressor protein menin.^4, 9, 10, 11^ Menin interacts with histone modifying proteins including the histone methyltransferases, mixed lineage leukaemia protein 1 (MLL1) and protein arginine methyltransferase 5 (PRMT5).^4, 9, 12, 13^ Furthermore, sporadic and familial PNETs have mutations of chromatin remodelling genes including death domain-associated protein (*DAXX*) and alpha thalassemia/mental retardation syndrome X-linked (*ATRX*),^14^ and sporadic BNETS have alterations of histone residues as well as increased expression of the histone methyltransferase enhancer of zeste homolog 2 (*EZH2*).^15^ These findings suggested that inhibitors targeting epigenetic pathways, may help in altering pro-oncogenic pathways in PNETs and BNETs, and we therefore assessed their *in vitro* and *in vivo* effects on NET proliferation and apoptosis.

## Results

### Effects of epigenetic pathway inhibitors on NET cell line proliferation

**Figure 1:**
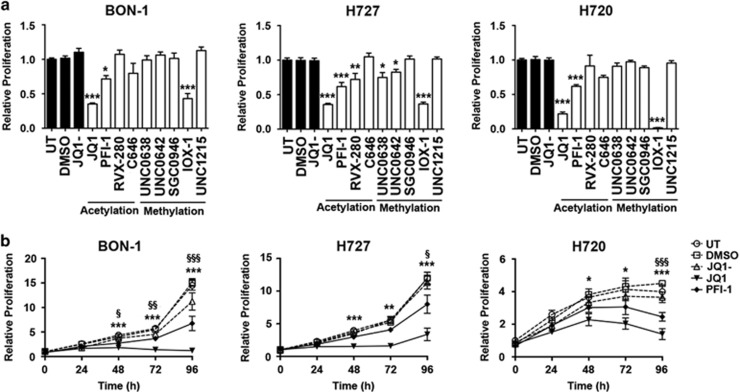
Efficacy of epigenetic pathway inhibitors in NET cell lines. The efficacy of epigenetic pathway inhibitors was examined in three NET cell lines BON-1 (derived from human metastatic PNET), H727 (derived from human BNET) and H720 (derived from human BNET). BON-1, H727 and H720 cells were treated with nine different compounds known to inhibit the activity of proteins (Supplementary Table S1) associated with histone modification or function; type of histone modification is indicated on the X axis (**a**). Proliferation was measured, using CellTiter Blue assays, 96 h after treatment with 1 μM of each compound (except IOX-1 which was used at 100 μM). For each experiment untreated (UT), vehicle only (DMSO) and JQ1- (a negative stereoisomer of JQ1) were used as negative controls. Control treatments are represented by black bars and test compound treatment by white bars. The efficacy of the BET inhibitors JQ1 and PFI-1 was further examined in BON-1, H727 and H720 NET cells. A 5 day timecourse of NET cell proliferation after a single treatment with 1 μM JQ1 or PFI-1, was also performed (**b**). Cells were treated with 1 μM JQ1-, JQ1 or PFI-1 and proliferation measured every 24 h. Control treatments are represented by dashed lines, and drug treatments by solid lines. For all experiments statistical significance relative to DMSO vehicle only treatment was assessed using a one-way ANOVA with (**a**) *P<0.05, **P<0.005 and ***P<0.0005, and in (**b**) PFI-1 statistics represented by the symbol §, and JQ1 statistics by * ^§/^*P<0.05, ^§§/^**P<0.005 and ^§§§/^***P<0.0005. For all experiments (**a**, **b**) untreated (UT), vehicle only (DMSO) and JQ1- were used as negative controls, and experiments performed in *n*=4, with 4 technical replicates per experiment.

We selected 9 inhibitors ((+)-JQ1 (JQ1), PFI-1, RVX-280, UNC0638, UNC0642, SGC0946, IOX-1, UNC1215 and C646) that target different components of epigenetic pathways for study. Five of these (UNC0638, UNC0642, SGC0946, IOX-1 and UNC1215) targeted histone methylation pathways, and the other 4 (JQ1, PFI-1, RVX-280 and C464) targeted histone acetylation pathways (Supplementary Table S1). We studied the *in vitro* effects of these 9 compounds on cell proliferation (by CellTitre Blue assay) of the PNET derived cell line (BON-1) and BNET cell lines (H727 and H720), all of which were found to not harbour any MEN1 mutations, consistent with previously reported data.^16, 17^ The concentration of each compound that was used was based on the available data of the dose required to yield a 90% effect (i.e. maximal inhibitory concentration (IC90)), which was 0.1–1 μM for most compounds, except IOX-1 that had an IC90 of 50–100 μM (Supplementary Table S1). Three of the compounds (JQ1, PFI-1 and RVX-280) targeting acetylated histone residues and 3 of the compounds (UNC0638, UNC642 and IOX-1) targeting methylated histone residues significantly reduced proliferation by 18–98% (*P*<0.05 to *P*<0.0001) of at least one NET cell line, when compared to NET cells treated with the negative control, inactive stereoisomer (JQ1-), or dimethyl sulfoxide (DMSO), or with untreated (UT) cells (Figure 1a). Importantly, the acetylation-targeting JQ1 and PFI-1, and the methylation-targeting IOX-1, significantly reduced proliferation of all 3 NET cell lines (Figure 1a), as follows: JQ1 reduced proliferation by 75, 65 and 78% in BON-1 cells (*P*<0.0005), H727 cells (*P*<0.0005) and H720 cells (*P*<0.0005), respectively; PFI-1 reduced proliferation by approximately 40% in all 3 NET cell lines (for H727 and H720 *P*<0.0005, and for BON-1 *P*<0.05); and IOX-1 reduced proliferation by 57, 64 and 98% in BON-1 cells (*P*<0.0005), H727 cells (*P*<0.0005) and H720 cells (*P*<0.0005), respectively. However, IOX-1, which is a pan-oxoglutarate oxygenase inhibitor that targets the Jumonji C domain family of histone lysine demethylases, had a >100 fold higher IC90, when compared to JQ1 (Supplementary Figure 5 and Supplementary Table 1), and this may partly be due to its low cell permeability, or the high intracellular concentration of oxoglutarate oxygenases.^18, 19^ However, the higher doses of IOX-1, that would potentially be required to yield the same efficacy as that for JQ1 and PFI-1, are likely to be associated with more off-target effects, and we therefore selected to further evaluate JQ1 and PFI-1, which were both effective and targeted the same pathway, namely histone acetylation.

### Evaluation of JQ1 and PFI-1 on NET cell line proliferation, cell cycle progression and apoptosis

**Figure 2:**
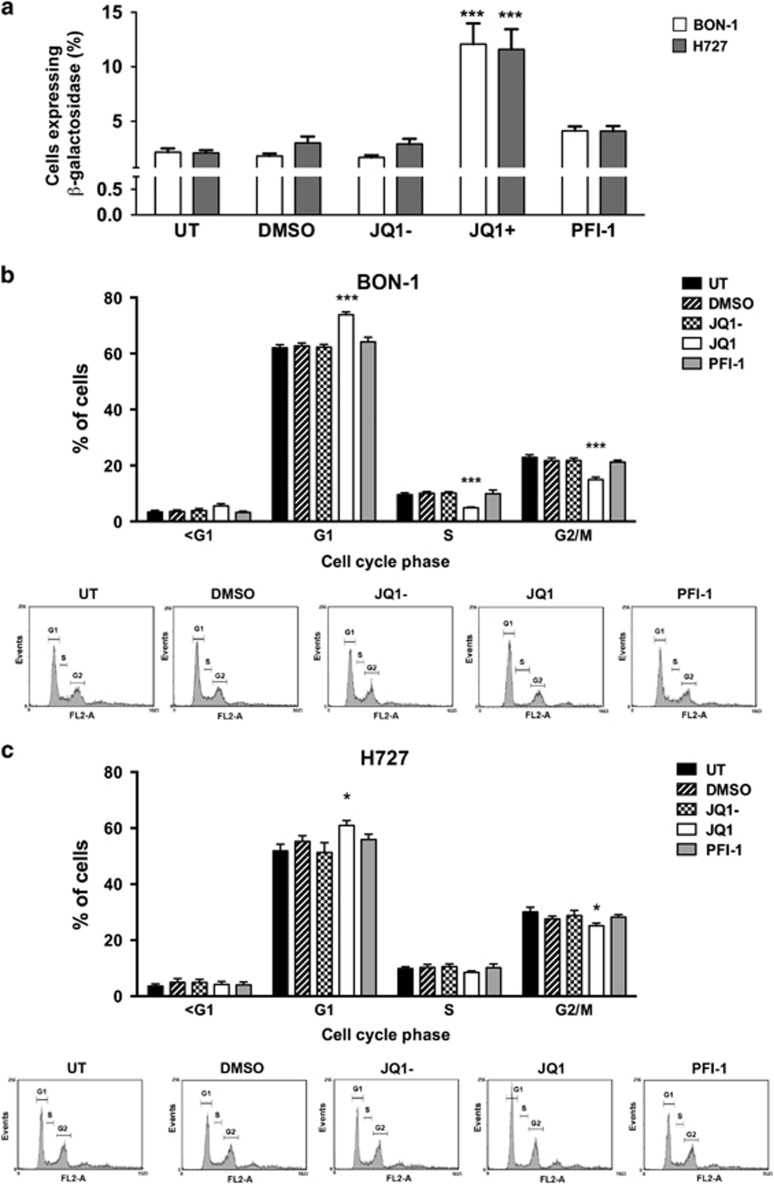
Effect of BET protein inhibition on cell cycle progression of NET cells. BON-1 and H727 cells were treated with a single dose of 1 μM JQ1 or PFI-1 compounds, and untreated (UT), vehicle only (DMSO) and JQ1- (a negative stereoisomer of JQ1) were used as negative controls. (**a**) The level of cellular senescence was measured, 96 h after compound treatment by determining the percentage of cells staining positively for β-glalactosidase. BON-1 cells are represented by white bars and H727 by grey bars. BON-1 (**b**) and H727 (**c**) cell cycle profiles were analysed after 48 h compound treatment using propidium iodide staining and flow cytometry. UT is represented by black bars, DMSO represented by bars with diagonal lines, JQ1- represented by chequered bars, JQ1 by white bars and PFI-1 treatment by grey bars. Gating for cell cycle stages is indicated on the histograms, with cells with lower fluorescence than the G1 gate classified as <G1. All experiments were performed in *n*=4, with 4 technical replicates per experiment, with statistical significance relative to DMSO control assessed using a one-way ANOVA *P<0.05; ***P<0.0005.

**Figure 3:**
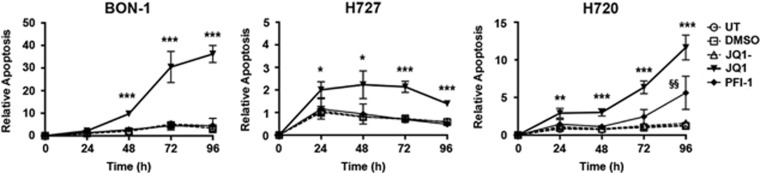
Efficacy of JQ1 and PFI-1 on apoptosis. Apoptosis of BON-1, H727 and H720 NET cell lines was evaluated every day for 5 days after a single dose of 1 μM JQ1 or PFI-1. Apoptosis was measured every 24 h after compound treatment by detecting caspase 3/7 activity. For all experiments untreated (UT), vehicle only (DMSO) and JQ1- were used as negative controls, and experiments performed in *n*=4, with 4 technical replicates per experiment. For all experiments statistical significance relative to DMSO vehicle only was assessed using a one-way ANOVA. PFI-1 results are represented by the symbol §, and JQ1 results by * ^§/^*P<0.05, ^§§/^**P<0.005 and ^§§§/^***P<0.0005.

JQ1 and PFI-1 target the bromodomains of the bromo and extra terminal domain (BET) protein family, which bind acetylated histone residues,^20^ and their effects on proliferation and apoptosis of the NET cell lines were investigated further. Proliferation time-course experiments showed that a single dose of JQ1 (1 μM) and PFI-1 (1 μM) at 48 and 96 h post-treatment, respectively, significantly reduced proliferation of all 3 NET cell lines by 1.5–2.6 fold (*P*<0.05 to *P*<0.0005) (Figure 1b), when compared to JQ1- or DMSO treated cells, or UT cells. Furthermore, both JQ1 and PFI-1 significantly decreased the proliferation of BON-1, H727 and H720 in a dose dependent manner (Supplementary Figure S1), with 1 μM treatment showing the greatest efficacy for both compounds (*P*<0.0005); and with the IC50 values for JQ1 and PFI-1, being 57–120 nM and 930–998 nM, respectively (Supplementary Figure S1). One μM of JQ1 and PFI-1, whose anti-proliferative effects continued for 72–96 h after removal of the compound (Supplementary Figure S2), was therefore used to assess the *in vitro* effects of JQ1 and PFI-1 on cell cycle progression and apoptosis. Cell cycle analysis showed that JQ1, but not PFI-1 treatment, significantly increased the percentage of senescent BON-1 and H727 cells (*P*<0.0005) (Figure 2a), and the percentage of BON-1 and H727 cells in G1 cell cycle stage (*P*<0.0005 and *P*<0.05, respectively) (Figure 2b and c). JQ1, but not PFI-1, treatment also significantly decreased the percentage of BON-1 and H727 cells in S and G2 cell cycle phases (*P*<0.05-*P*<0.0005). Moreover, JQ1 treatment at 48 h, also significantly increased apoptosis by ~4.5 fold, ~3 fold and ~2 fold in BON-1 (*P*<0.0005), H727 (*P*<0.05) and H720 cells (*P*<0.05), respectively, and by ~36 fold in BON-1 cells at 96 h (*P*<0.0005), whereas PFI-1 increased apoptosis of only H720 cells by ~5 fold after 96 h (*P*<0.005), when compared to cells treated with JQ1- or DMSO, or UT cells (Figure 3). Thus, JQ1 was more potent than PFI-1 in inhibiting proliferation and cell cycle progression, and in increasing apoptosis in the 3 NET cell lines.

### JQ1 increases histone 2B (H2B) expression in NET cell lines

**Figure 4:**
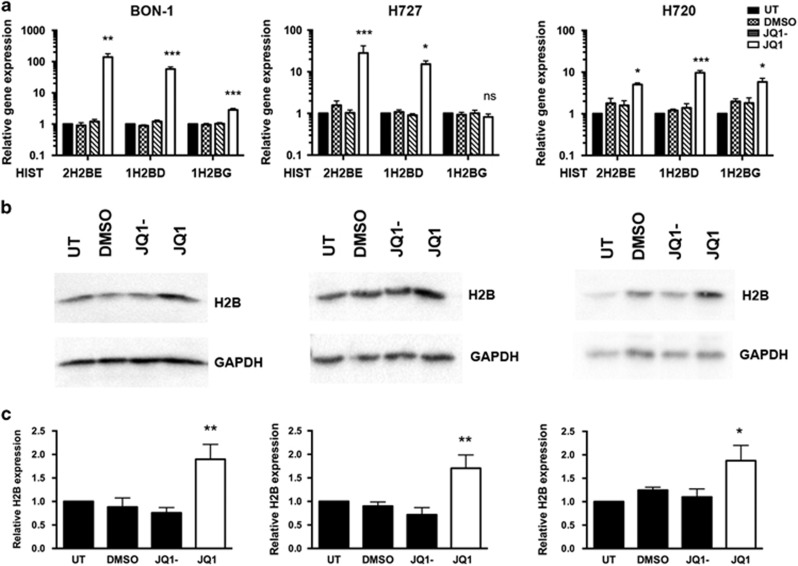
Mechanism of action of JQ1 in NETs. RNA sequencing (RNA-Seq) was performed in treated (JQ1) and control (JQ1-, untreated and DMSO) human NET cell lines BON-1, H727 and H720 and changes in gene and protein expression were assessed by qRT-PCR and Western blot analysis, respectively. (**a**) Expression of significantly dysregulated histone genes detected by RNA-Seq was examined by qRT-PCR. Untreated (UT) is represented by a black bar, DMSO control treatment by chequered bars, JQ1- control treatment by lined bars, and JQ1 by white bars. A one-way ANOVA, was used for statistical analsyis, *P<0.05; **P<0.005; ***P<0.0005. Data is represented relative to untreated cells. (**b**) Western blot analysis of H2B protein expression in JQ1 and control treated BON-1, H727 and H720 cells. GAPDH was used as a loading control. (**c**) Quantification of H2B Western blot analsyis using densitometry. Control treatments are represented by black bars and JQ1 treatment by white bars. One-way ANOVA using a Bonferroni correction for multiple comparisons was used for statistical analysis,*P<0.05; **P<0,005. Data and significance is represented relative to UT cells.

To determine the possible mechanisms of action of JQ1 in human NET cells, RNA sequencing (RNA-Seq) was performed on BON-1, H727 and H720 cell lines treated with JQ1 or JQ1-, for 48 h. This showed that JQ1 treatment resulted in: 1945 up-regulated and 1716 down-regulated genes in BON-1 cells; 816 up-regulated and 1434 down-regulated genes in H727 cells; and 693 up-regulated and 1469 down-regulated genes in H720 cells (all >2 fold change, *P*<0.001). Comparison of these genes showed that 283 genes and 127 genes were commonly up-regulated and down-regulated in all 3 cell lines, and analysis of these genes indicated that JQ1 significantly dysregulated 22 canonical pathways (Supplementary Table S2), thereby suggesting that the likely mechanism of action of JQ1 in NETs is through multiple genes and pathways. However, 6 of the top 20 most highly significantly up-regulated genes, after JQ1 treatment, were involved in histone (HIST) complex formation, with *HIST2H2AA3*, *HIST1H2AC* and *HIST2H2AG* encoding histone (H)2A protein isoforms, and *HIST2H2BE*, *HIST2H2BD* and *HIST1H2BC* encoding H2B protein isoforms (Supplementary Table S3). Furthermore, there were 5 H2A and 10 H2B genes up-regulated in BON-1 cells, 3 H2A and 10 H2B genes up-regulated in H727 cells, and 2 H2A and 3 H2B genes up-regulated in H720 cells, which also included *HIST1H2BG* and *HIST1H2BF*. Quantitative real-time PCR (qRT-PCR) was used to confirm expression of 4 of these highly up-regulated histone H2B genes and 4 highly downregulated genes (*sarcoma proto-oncogene, non-receptor tyrosine kinase* (*SRC*), *DNA fragmentation factor subunit beta* (*DFFB*), *Inhibin beta E subunit* (*INHBE*) and *HEPACAM family member 2* (*HEPACAM2*)). Thus, *HIST2H2BE* and *HIST1H2BD* were significantly up-regulated in all three NET cell lines and *HIST1H2BG* was up-regulated in BON-1 and H727 cells after JQ1 treatment, when compared to cells treated with JQ1- or DMSO, or UT cells (Figure 4a); and *SRC*, *DFFB*, *INHBE* and *HEPACAM2* were significantly downregulated in all 3 NET cell lines (Supplementary Figure S3). Specific antibodies for the proteins encoded by *HIST2H2BE, HIST1H2BD* and *HIST1H2BG* genes are not available, and we therefore assessed their combined expression by assessing total histone (H)2B expression by Western blot analysis. This confirmed that total H2B protein expression was increased, in all 3 NET cell lines, after JQ1 treatment, when compared to control treatments (Figure 4b and c), thereby indicating that JQ1 treatment may alter H2B abundance.

### *BRD2* is the most abundant BET family member in NET cell lines and PNETs of *Men1*^*L/L*^*/RIP2-Cre* mice

**Figure 5:**
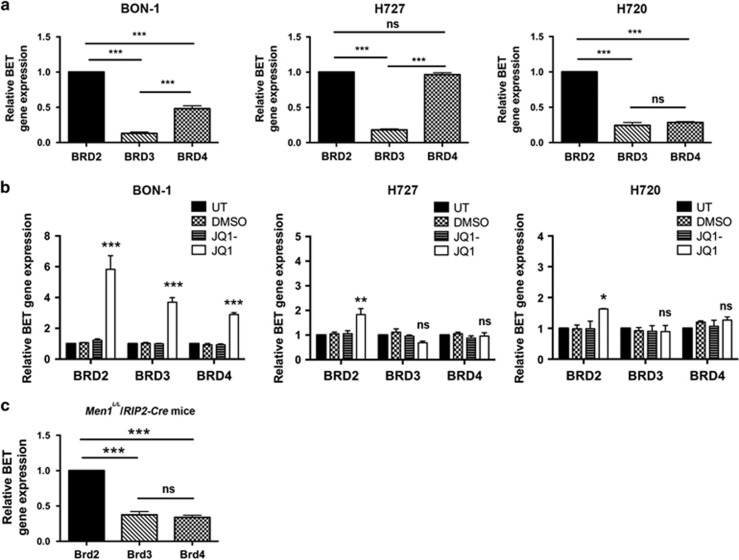
BET gene expression in NET cell lines and murine PNETs. (**a**) Expression of the BET protein family members *BRD2*, *BRD3* and *BRD4* was examined in BON-1, H727 and H720 NET cell lines using qRT-PCR. All data is expressed relative to BRD2 expression. ***P<0.0001; ns – not significant; *n*=4 biological replicates. (**b**) Relative BET expression in BON-1, H727 and H720 cell lines after JQ1 treatment, or control untreated (UT), DMSO or JQ1- treatment. Significance is relative to UT cells; ns—not significant, *P<0.01, **P<0.001, ***P<0.0001; *n*=4 biological replicates. (**c**) BET expression in PNETs isolated from female (*n*=4) and male (*n*=4) untreated 34 week old *Men1*^*L/L*^*/RIP2-Cre* mice. BET gene expression was similar in male and female mice. ***P<0.0001; ns—not significant. Statistical significance was assessed using a one-way ANOVA.

JQ1 is reported to act through BRD4 in decreasing expression of the oncogene *c-myc*.^21, 22, 23^ However, our RNA-Seq analysis, which aimed to assess simultaneous alterations of gene expression in the BON-1, H727 and H720 cells after JQ1 treatment, did not find significant down-regulation of *c-myc*. This suggested that JQ1 may act via other mechanisms in NET cells, and this is likely to be the case for H720 cells which were found, by Western blot analysis, not to express *c-myc* (Supplementary Figure S4). We therefore hypothesised that JQ1 may act via BET family members and other target proteins, in NETs. Indeed, our examination of the RNA-Seq data revealed an increase in *BRD2* expression, by 2.27-fold and 2.05-fold in BON-1 and H727 cells, respectively. We therefore examined the expression of the BET family, which consists of 4 members *BRD2*, *BRD3*, *BRD4* and the testes-specific *BRDT*,^24^ in the 3 NET cell lines by qRT-PCR to confirm the findings from the RNA-Seq data. This revealed that *BRD2* was the most abundant BET family member in the 3 NET cell lines, with *BRD2* expression being significantly higher than that of *BRD3* and *BRD4* in BON-1 and H720 cells (2.1–7.7 fold, *P*<0.0005, for both), and in H727 cells being significantly higher expression than that of *BRD3* (5.6 fold, *P*<0.0005) but equal to that of *BRD4* (Figure 5a). *BRD3* was the least abundant BET family member in all 3 NET cell lines, with *BRD4* expression being variable (Figure 5a); *BRDT*, which is testes-specific, was not expressed. JQ1 treatment, when compared to treatment with JQ1- or DMSO, or no treatment, significantly increased *BRD2* expression in BON-1, H727 and H720 NET cell lines by 5.8 fold (*P*<0.0001), 1.8 fold (*P*<0.001) and 1.6 fold (*P*<0.01), respectively (Figure 5b). JQ1 treatment, compared to controls, also significantly increased *BRD3* and *BRD4* expression by 3.6 fold (*P*<0.0001) and 2.8 fold (*P*<0.0001), respectively in BON-1 cells; however, *BRD2* expression was significantly higher than that of *BRD3* and *BRD4,* by 2.2 fold (*P*<0.001) and 3 fold (*P*<0.001), respectively, in BON-1 cells (Figure 5b). JQ1 treatment did not affect *BRD3* and *BRD4* expression in the BNET cells H727 or H720 (Figure 5b). *Brd2* expression was also significantly higher than that of *Brd3* (*P*<0.0005) and *Brd4* (*P*<0.0005) in PNETs that developed in *Men1*^*L/L*^*/RIP2-Cre* mice (Figure 5c), thereby suggesting that the expression of these BET family members in the NET cell lines is representative of that occurring *in vivo* in PNETs of *Men1*^*L/L*^*/RIP2-Cre* mice.

### Efficacy of the BET bromodomain inhibitor JQ1 on PNET growth *in vivo*

**Figure 6:**
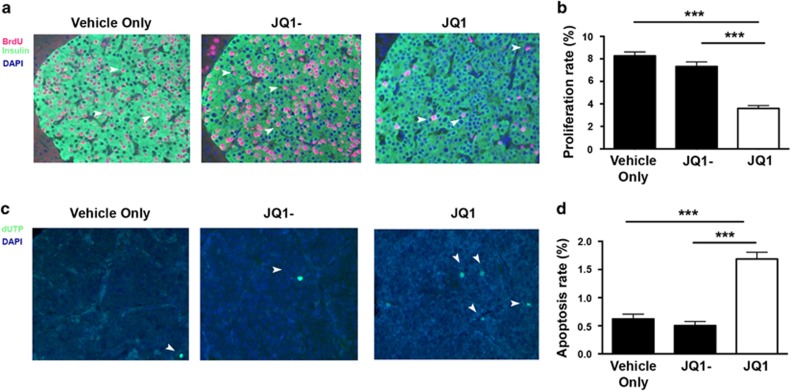
Efficacy of JQ1 on proliferation and apoptosis of pancreatic NETs in *Men1*^*L/L*^*/RIP2-Cre* mice. Thirty week old *Men1*^*L/L*^*/RIP2-Cre* mice (*n*=8; 4 males and 4 females) were injected, i.p., with JQ1 twice weekly for one month, and the pancreas removed to study PNET growth. (**a**) Immunohistochemistry of bromodeoxyuridine (BrdU) incorporation into proliferating PNET cells. BrdU stained cells are indicated in red and by the white arrows. PNETs were counterstained with DAPI (blue) to highlight the nucleus of individual cells, and insulin (shown in green) to define the tumor size. (**b**) Apoptosis in PNETs were detected using TUNEL assay. Apoptotic cells are indicated by the addition of labelled dUTP (green, and indicated by white arrows). All sections were counterstained with DAPI to detect individual cells and tumor areas were defined by analysis of serial sections stained for insulin. All images are at x200 magnification. (**c**) Only cells co-staining for BrdU and insulin were included for quantification analysis of BrdU incorporation. Proliferation rate was determined as percentage of BrdU and insulin immunostaining per tumor, with *n*=3 tumors counted from *n*=4 sections per mouse. *P<0.05 and **P<0.005. Control treatments are indicated by black bars, and JQ1 treatment by a white bar. (**d**) Quantification of apoptosis rates. Apoptosis rate was calculated as the percentage of dUTP positive cells per tumor, with *n*=3 tumors counted from *n*=4 sections per mouse. ***P<0.0005. Control treatments are indicated by black bars, and JQ1 treatment by a white bar. The efficacy of JQ1 in male and female mice was the same. Statistical significance was assessed using a one-way ANOVA.

The *in vivo* efficacy of JQ1 as a treatment for PNETs due to loss of menin expression (Supplementary Figure S5) was evaluated by twice weekly intraperitoneal (i.p.) injection of 50mg/kg of JQ1, JQ1- or vehicle only to *Men1*^*L/L*^*/RIP2-Cre* mice, with the doses and administration frequency based on previous studies^20, 22^ and our *in vitro* data (Supplementary Figure S2). Proliferation (Figure 6a) and apoptosis (Figure 6c) in PNETs from *Men1*^*L/L*^*/RIP2-Cre* mice were assessed after one month of treatment, but tumour dimensions were not assessed, as the short duration of the study was unlikely to result in measurable changes to this parameter of tumour burden. The proliferation rate of PNETs from JQ1 treated mice was significantly lower at 3.7%, in comparison to the proliferation rates of 7.1% (*P*<0.0005) and 8.1% (*P*<0.0005), in JQ1- and vehicle only treated mice, respectively (Figure 6b). Thus, JQ1 reduced the proliferation rate of PNETs by 49%-55%. JQ1 treatment also significantly increased the apoptosis rate of PNETs by 3.2 fold (*P*<0.0005) and 2.8 fold (*P*<0.0005), when compared to JQ1- and vehicle only treated mice, respectively (Figure 6d).

## Discussion

Our studies demonstrate that JQ1 treatment can significantly reduce proliferation and increase apoptosis of human PNET and BNET cell lines, and of mouse PNETs *in vivo*. In addition, our results show that the BET family of proteins and regulation of histone expression are important mechanisms for NET growth, and that BET inhibitors, such as JQ1, may provide novel therapeutic drugs for the treatment of NETs.

JQ1, PFI-1 and RVX-280, which all had anti-proliferative effects on NET cells (Figure 1), represent compounds that specifically target and inhibit the bromodomains of the BET family proteins (BRD2, BRD3, BRD4 and BRDT) (Supplementary Table S1). BRD proteins bind to acetylated lysine residues on histone tails through their 2 bromodomains to activate transcription of target genes, for example by the recruitment of the positive transcriptional elongation factor b (P-TEFb) by BRD4, and the interaction of BRD2, BRD3 and BRD4 with the transcription factor GATA1.^24, 25, 26^ JQ1 and PFI-1, which target the N- and C-terminal bromodomains, had greater effects and significantly reduced proliferation by 40–95% (Figure 1), in all 3 NET cell lines. Moreover, JQ1 increased apoptosis by up to 36 fold in all 3 NET cell lines, whereas PFI-1 increased apoptosis by only 5 fold in one NET cell line (Figure 3). RVX-280, which primarily targets the C-terminal bromodomain, that is reported not to be associated with anti-proliferative effects^27^ had the least efficacy and significantly reduced proliferation in only 2 of the 3 NET cell lines (Figure 1). Thus, our results suggest that BET family N- and C- terminal bromodomains may be important for exerting tissue-specific actions on proliferation and apoptosis, and therefore that both bromodomains are likely involved in acetyl-lysine binding activity. These findings are consistent with those of a recent study that reported that the BET bromodomain inhibitor, CP1203, reduces proliferation of BON-1 and H727 cells, although no effects on apoptosis were observed; that JQ1 and PFI-1 may also alter proliferation of BON-1 cells and H727 cells; and that JQ1 may alter cell cycle progression.^28^ The variations in the *in vitro* efficacy of different bromodomain inhibitors in NET cells, may also be due to differences in cell permeability, solubility and bioavailability.^27, 29, 30^ Our results also show that these *in vitro* mechanisms are likely to be important *in vivo*, as our data demonstrated that JQ1 could significantly reduce proliferation and increase apoptosis of PNETs that developed in a MEN1 mouse model (Figure 6). Thus, our results expand the spectrum of tumors that have been effectively targeted in pre-clinical studies, by bromodomain inhibitors, such as JQ1 and iBET151, and this includes solid tumors (e.g. pancreatic ductal carcinoma and nuclear in testes (NUT)-midline carcinoma) and leukaemias.^28, 31, 32, 33, 34^ Moreover, some of these pre-clinical findings have advanced to clinical trials aimed at evaluating the efficacy of bromodomain inhibitors in treating various tumor types including NUT midline carcinoma, acute myeloid leukaemia and myelomas (ClinicalTrials.gov).

Our studies have revealed an important role for *BRD2* in NET cell lines and mouse PNETs. Thus, *BRD2* was expressed more highly than *BRD3* and *BRD4* in NET cell lines (Figure 5a and b) and mouse PNETs (Figure 5c), and JQ1 had greater effects on *BRD2* expression than on *BRD3* and *BRD4* expression (Figure 5b). These findings are consistent with BET family members having discrete roles in pancreatic β-cells, with BRD2 reported to have a role in insulin secretion,^35^ and to induce insulin resistance by enhancing signalling mediated by the mechanistic target of rapamycin (mTOR) and phosphoinositide 3-kinase (PI3K).^36^ Interestingly, our RNA-Seq data indicated potential changes in mTOR and PI3K signalling (Supplementary Table S2), thereby suggesting possible roles of BRD2 and mTOR signalling in PNETs and BNETs. However, our results are in contrast to findings from studies of other tumours (e.g. medulloblastoma, hepatocellular carcinoma and acute leukaemia), and in BON-1 cells, in which the primary target of JQ1 has been reported to be BRD4 that has roles in tumour development by transcriptional regulation of tumour promoting proteins such as c-myc,^21, 22, 23, 28, 37, 38^ and in apoptosis by activation of p53.^39^ In addition, treatment of BON-1 cells by the BET inhibitor CP1203 caused a down regulation of c-Myc.^28^ Thus, in these studies, the key actions of BET inhibition are via reduced c-myc transcription and activation of p53. However, it seems likely that in other tumours, and cancer cells, that JQ1 may act by different mechanisms, especially if these genes are not expressed in the tumour cells, as occurs in BNET H720 cells, which we found to not express c-myc (Supplementary Figure S4). Furthermore, H727 cells have a reported single nucleotide polymorphism (SNP) in *BRD4*, and there remains a possibility that this could alter its expression and response to drugs such as JQ1. In addition, use of sub-clones of these cell lines, that may have acquired different SNPs, may also account for differences in their responses due to altered BET protein function.^16, 17^ However, it is important to note that JQ1 has been reported to be effective in NUT-midline carcinoma without altering c-myc expression,^40^ which is similar to our findings from RNA-Seq analysis of JQ1 treated NET cell lines, that did not detect any changes in p53 or c-myc expression. These findings therefore suggest that JQ1 may reduce NET tumour growth by different mechanisms.

In NETs such mechanisms may involve dysregulation of histone expression, as reported in other cancers, which have altered abundance of H2B protein isoforms that may disrupt their critical roles in DNA packaging and lead to chromosomal abnormalities during cell division that prevents cell cycle progression and leads to cell death.^41^ Moreover, our results in NET cells have shown that JQ1 causes a significant increase in expression of *HIST2H2BE*, *HIST2HBD* and *HIST2H2BG* (Figure 4), thereby indicating that the BRD proteins may regulate H2B expression. However, the role of the BRD proteins in regulating histone expression is complex with: BRD2, BRD3 and BRD4 reported to be present at the promoter of histone genes such as *HIST2H2BE*, *HIST2H2AA* and *HIST2H2AB* in HeLa cells^42^ and BRD2 regulating activity of the histone variant H2A.Z in embryonic stem cells.^43^ In addition, BRD proteins have also been reported, by ChiP-Seq analysis, to bind to regions of their own promoter and to each others’ promoters,^42^ and thus it seems possible that changes in *BRD2*, *BRD3* and *BRD4* mRNA expression after JQ1 treatment, as shown by our data (Figure 5), may alter BRD protein expression and function. Moreover, the precise roles of menin, and its loss, in regulating BRD proteins remains to be elucidated. However, menin can regulate gene transcription through changes in histone methylation and acetylation,^4^ and hence it seems possible that changes in menin expression may alter the level of histone acetylation, which could in turn lead to increased BRD proteins at acetylated histones. However, JQ1 has been demonstrated to have efficacy in a number of different tumour types which do not show alterations in menin expression,^28, 31, 32, 33, 34^ and DNA sequence analysis of the NET cell lines in our study, revealed that they do not have mutations in the *MEN1* gene. It is therefore likely that BRD proteins may act through generic pathways, including alterations in histone expression to modulate proliferation and apoptosis.

Our findings, which reveal that JQ1 treated NET cells stall at the G1 phase of the cell cycle, with a reduction of cells in the S and G2 phases (Figure 2), suggest that such regulation of histone expression may be important in the cell cycle of tumor cells. The cell cycle specific regulation of the histone genes and their protein isoforms, in tumour cells, remains to be elucidated. However, in normal cells histone levels fluctuate during different stages of the cell cycle, with the expression of key histones restricted to the S-phase, where they are incorporated into DNA.^44^ There are over 20 H2B genes, located in 3 genetic clusters that encode over 13 different protein isoforms,^41^ and *HIST2H2BE*, *HIST2HBD* and *HIST2H2BG,* whose expression is increased by JQ1, are all expressed from cluster 2 on chromosome 1q21.2. *HIST2H2BE*, *HIST2HBD* and *HIST2H2BG* encode core replication-dependent histones which are expressed during S-phase, and incorporated into the nucleosomes in replicating DNA,^41^ and the H2B gene promoter contains subtype-specific regulatory elements and a TATA-box, with the Oct-1 protein acting through these elements to promote transcription.^45^ However, the S-phase specific expression of H2B is regulated by the transcriptional co-activator, OCA-S, which interacts with the cycinE/cdk2 substrate, NPAT.^44, 45^ Moreover, the cyclin E/cdk2 complex and a histone deacetylase complex containing surtuin 1 stimulate the histone acetyl transferase CBP/p300, which regulates acetylation of H3 tails at the promoter regions of the histone genes that fluctuates during the cell cycle, peaking at the G1/S phase transition. The BET protein family has key roles in these processes as they regulate the expression of cyclin proteins, including regulation of cyclin E expression by BRD2, and bind to acetylated residues on histone tails.^24, 46^ Thus, it seems possible that JQ1 treatment of NET cells and inhibition of BET protein activity, may disrupt the cell cycle-specific regulation of H2B, either directly through binding of BRD proteins to the histone promoters, or through regulation of cell cycle components, thereby leading to increased expression of histone proteins.

In conclusion, our studies show that the BET inhibitor JQ1 can reduce proliferation and increase apoptosis of NET cells *in vitro* and pancreatic NETs *in vivo*, thereby indicating the potential utility of BET inhibitors in the treatment of NETs.

## Materials and methods

### Cell lines, *In vitro* assays and compounds

BON-1 cells^47^ and H727 cells (ATCC ♯CRL-5815) were cultured in DMEM-F12 and RPMI media (Gibco, Paisley, UK), respectively, supplemented with 10% fetal calf serum (FCS) (Sigma-Aldrich, St Louis, MO, USA). H720 cells (ATCC ♯CRL-5838) were cultured in DMEM-F12 supplemented with 5% FCS, 10 nM hydrocortisone, 10 nM β-estradiol and 1x ITS (containing insulin, transferrin and selenous acid) (Sigma-Aldrich). All cells were maintained at 37 °C, 5% (vol/vol) CO_2,_ and tested for mycoplasma using the MycoAlert kit (Lonza, Basel, Switzerland). Untreated and DMSO only treated cells were used as controls. Cell proliferation was assessed using CellTitre-Blue Cell Viability assay (Promega, Madison, WI, USA) whereby 20 μl of CellTitre-Blue reagent was added per well, incubated for 2 h at 37 °C, 5% (vol/vol) CO_2_, before fluorescent output read on a CytoFluor microplate reader (PerSeptive Biosystems, Paisley, UK) at 530 nm excitation and 580 nm emission.^48^ Apoptosis was evaluated using the CaspaseGlo 3/7 assay (Promega) whereby 75 μl of CaspaseGlo reagent was added per well, incubated for 1 h at room temperature, and luminescent output read on a Turner Biosystems luminometer.^48^ Cellular senescence was determined using the Cellular Senescence Assay (SA-β-gal staining) kit (Cell Biolabs, San Diego, CA, USA),^49^ 96 h after drug treatment. Cells were visualised using an Eclipse E400 microscope (Nikon, Tokyo, Japan), and images captured using a DXM1200C digital camera and NIS-Elements BR 2.30 software (both Nikon). The percentage of positively stained cells were counted, per total cells, from *n*=20 10x magnification fields. Cell cycle progression was determined using proidium iodide (PI) staining, 48 h after drug treatment. Cells were fixed in 100% ethanol, washed with PBS and DNA stained by incubation with PI solution (10 μg/ml PI, 200 μg/ml RNase, 0.1% Triton-X, 150 mM sodium chloride; diluted in PBS) for 30 min at room temperature. Intensity of PI staining was detected on a FACScalibur flow cytometer (Becton Dickinson, Franklin Lakes, NJ, USA) using Cellquest pro software. Peaks of fluorescence, representing DNA content, were used to evaluate the percentage of cells at each cell cycle stage, using FloJo software. The 9 compounds (JQ1, PFI-1, RVX-280, UNC0638, UNC0642, SGC0946, IOX-1, UNC1215 and C464), as well as one inactive control compound (JQ1-), were supplied by the Structural Genomics Consortium (SGC, Oxford) (Supplementary Table S1). All compounds were suspended/diluted in dimethyl sulfoxide (DMSO, Sigma-Aldrich); details of the concentrations used and the biological targets of each compound are provided in Supplementary Table S1.

### Quantitative real time PCR (qRT-PCR)

Total RNA was extracted from NET cell lines and mouse PNET tissues using the miRVana kit (Ambion), and 1 μg used to generate cDNA using the Quantitect Reverse Transcription kit (Qiagen), as described.^50^ Quantitect primers (Qiagen) were used for qRT-PCR reactions, which utilised the Quantitect SYBR green kit (Qiagen), on a RotorGene 5, as described.^50^ Each test sample was normalized to the geometric mean of reference genes GAPDH and α-tubulin. The relative expression of target cDNA in all qRT-PCR studies was determined using the Pfaffl method.^51^

### Western blot analysis

NET cells were prepared in NP40 lysis buffer: 250 mM NaCl, Tris 50 mM (pH 8.0), 5 mM EDTA, 0.5% NP-40 (v/v) and 2x Protease inhibitor tablets (Roche), as described.^50^ Lysates were prepared in 4x Laemmli loading dye (BioRad) boiled at 95 °C for 5 min, resolved using 6 or 10% SDS-PAGE gel electrophoresis, transferred to polyvinylidene difluoride membrane, and probed with primary antibodies (GAPDH–ab9485, and H2B-ab1790, c-myc-ab32072, all AbCam), anti-rabbit HRP-conjugated secondary antibody (Santa Cruz Biotechnology, Dallas, TX, USA) and visualised using Pierce ECL Western Blotting substrate (Thermo Fisher Scientific, Paisley, UK), as described.^50^ GAPDH protein expression was used as a loading control. Densitometry analysis was performed by calculating the number of pixels per band using ImageJ software. Data was represented as the number of pixels of the protein band, relative to the number of pixels of the corresponding GAPDH band.

### RNA sequencing and analysis

Confluent cells were treated with 1 μM JQ1- or JQ1 for 48 h, before RNA extraction using the RNeasy kit (Qiagen). RNA sequencing was performed on 3 independent experimental replicates for each cell line and treatment. Following polyA-enrichment and library preparation, 50 bp paired-end sequencing (Illumina 2500 machine) generated >25 million read-pairs per sample. Reads were aligned to the human reference genome (GRCh37) using TopHat2^52^ and duplicate reads removed (Picard Tools MarkDuplicates). ~20 million high-quality reads per sample were mapped uniquely to Ensembl-annotated genes; gene counts were summarised using HTSeq^53^ and filtered to exclude genes with fewer than 10 reads on average per sample. Data normalisation and differential expression analysis, comparing JQ1 and JQ1- in each cell line separately, was performed using the edgeR package.^54^ Genes with adjusted *P*-value <0.05 and showing a fold change >2 in either direction were considered significant. Identification of altered cellular pathways was undertaken using QIAGEN Ingenuity Pathway Analysis (IPA, QIAGEN, www.qiagen.com/ingenuity).

### *In vivo* studies

Mice were kept in accordance with UK Home Office welfare guidelines and project licence restrictions. *Men1*^*L/L*^/*RIP2-Cre* mice, which have a homozygous excision of *Men1* exons 3–8 in pancreatic β-cells and develop insulin-containing PNETs by 6 months of age,^55^ were generated by breeding 129S(FVB)-*Men1*^*tm1.2Ctre*^/J (*Men1*^*L/L*^) with B6.Cg-Tg(Ins2-cre)25Mgn/J (*RIP2-Cre*) (Jackson Laboratory USA). Genotyping was performed as described.^55^ Mice were fed a standard diet (Rat and Mouse No. 1 (RM1) expanded diet, Special Diet Services, Ltd) and provided with water *ad libitum*, which for 4 weeks before sacrifice, contained BrdU (1 mg/ml), to allow assessment of *in vivo* cell proliferation. Littermate 129S(FVB)-*Men1*^*tm1.2Ctre*^/J (*Men1*^*L/L*^) mice, i.e. mice expressing *Men1*^*L/L*^ but not *RIP2-Cre*, from breeding cohorts were used as controls. *Men1*^*L/L*^/*RIP2-Cre* mice were aged to 30 weeks before entering the study. From 30 weeks of age mice received for one month, a twice weekly i.p. injection of 50 mg/kg JQ1 or JQ1-, or vehicle only (DMSO diluted in 2-hydroxypropyl-β-cyclodextrin). All drugs were diluted in 20% aqueous solution of 2-hydroxypropyl-β-cyclodextrin. Eight mice (4 males and 4 females) were used in the study, and these sample sizes were determined using the ‘Resource Equation’ *E=N-B-T* as effect sizes were unknown, (*E=*degrees of freedom for the error term used to test the effect of the variable, and should be between 10–20; *N*=total number of mice-1; *B*=number of blocks-1; and *T*=number of treatment groups-1). No animals were excluded from the study. Randomization of the animals or blinding of the investigators to the procedures, which were not appropriate for this study, were not undertaken. Pancreatic tissues were dissected from mice, fixed with 4% paraformaldehyde and embedded in paraffin, as described.^56^ For immunohistochemistry 4 μM sections were dewaxed and hydrated, underwent heat-mediated antigen retrieval in citrate buffer and were blocked using 10% donkey serum before primary antibody incubation, as described.^56^ Primary antibodies (AbCam) included rabbit anti-menin (ab2605), rabbit anti-insulin (ab7842) and rat anti-BrdU (BU1/75 (ICR1)). Secondary antibodies included Cy2-conjugated donkey anti-rabbit (Jackson ImmunoResearch) and Cy3-conjugated donkey anti-rat (Jackson ImmunoResearch) or horseradish peroxidase-conjugated goat anti-rabbit antibody (Dako) with a peroxidase/3,3′-diaminobenzidine Envision detection system (Dako). Nuclear counterstaining was performed with 4′,6-diamino-2-phenylindole (DAPI) (ProLong Gold Antifade reagent with DAPI; Molecular Probes). Sections were viewed by light or fluorescent microscopy using an Eclipse E400 microscope (Nikon), utilising a DXM1200Cdigital camera and NIS-Elements BR 2.30 software (both Nikon), as described.^56^ Cell proliferation and apoptosis rates were obtained from an average of 12 x10 magnification fields per animal and *n*=8 (*n*=4 female and *n*=4 male) mice were examined in each treatment group. Daily cellular proliferation rate was determined by calculating the percentage of BrdU positively stained cells and dividing by the number of consecutive days of BrdU administration, as described.^56^ Apoptotic rate was determined using the ApopTag Fluorescein Direct *In Situ* Apoptosis Detection Kit (Millipore), which utilises terminal deoxynucleotidyl transferase (TdT) to detect and label free 3′OH DNA termini caused by fragmentation, as described.^56^

### Statistical analysis

Data were analysed using one-way analysis of variance (ANOVA) using a Bonferroni correction for multiple comparisons, or Dunnett’s test for a single control group, as previously described.^50, 56^ For *in vitro* data comparisons are relative to DMSO control, and for RNA-Seq and *in vivo* data comparisons are relative to JQ1- treatment. Data from the *in vitro* and *in vivo* experiments were found to have a normal distribution, with similar variance between the compared groups. RNA-Seq data were analysed using statistical models utilising in the edgeR package^54^ and Benjamini-Hochberg’s method applied to control the false discovery rate.^57^
