# Supplementary material for: Epigenetic pathway inhibitors represent potential drugs for treating pancreatic and bronchial neuroendocrine tumors
Source: Oncogenesis. 2017 May 15;6(5):e332–. doi: 10.1038/oncsis.2017.30 (PMC5523063; doi:10.1038/oncsis.2017.30)
Supplement: Supplementary Information [file oncsis201730x1.docx]

**Supplementary Data**

**Supplementary Table S1**. Epigenetic modification targeting compounds, their protein targets and concentrations used for *in vitro* assays.

**Supplementary Table S2.** Ingenuity pathway analysis of genes commonly dysregulated in BON-1, H727 and H720 cells after JQ1 vs JQ1- treatment. Only significantly altered pathways are shown (p<0.05).

**Supplementary Table S3.** The 20 most highly up- or down-regulated genes after RNA-sequencing of JQ1 treated human cell lines. Fold change values (LogFC) are represented as a logarithm to the base 2, as JQ1 versus JQ1- treatment. LogFC values also denote the average fold change in gene expression across all 3 cell lines (BON-1, H727 and H720) investigated. Genes were significantly dysregulated, p<0.001.

**Supplementary Figure S1.** **Dose response curves of JQ1 and PFI-1 BET inhibitors in NET cell lines**. BON-1, H727 and H720 NET cell lines were treated with increasing doses (20nM-1μM) of JQ1 and PFI-1 compounds for 96h. Proliferation was measured and compared to the proliferation of inactive control compound, JQ1-, treated cells. IC50 values are shown in red. JQ1 statistics are represented by * and PFI-1 statistics by §. §/* p<0.05; §§/** p<0.005; §§§/*** p<0.0005, as indicated by one-way ANNOVA.

**Supplementary Figure S2.** **Proliferation of NET cells after compound removal.** BON-1 and H727 cells were treated with 1μM JQ1-, JQ1 or PFI-1 compounds for 96h; untransfected (UT) and DMSO treatment were also included as controls. After 96h compound-containing media was replaced with regular culture media and proliferation measured every 24h for 5 days. PFI-1 statistics are represented by the symbol §, and JQ1 statistics by *; §/* p<0.05, §§/** p<0.005 and §§§/*** p<0.0005, as indicated by one-way ANNOVA. Significance is relative to DMSO vehicle only treatment.

**Supplementary Figure S3. Validation of RNA-Seq data.** Expression of genes significantly down regulated by RNA-Seq was examined by qRT-PCR in BON-1, H727 and H720 cell lines. The expression of *sarcoma proto-oncogene, non-receptor tyrosine kinase* (*SRC*), *DNA fragmentation factor subunit beta* (*DFFB*), *Inhibin beta E subunit* (*INHBE*) and *HEPACAM family member 2* (*HEPACAM2*) were chosen as they were down-regulated by 2.07-fold, 2.16-fold, 13.20-fold, and 21.26-fold, respectively in the RNA-Seq data, and have cancer, or NET related functions. Thus the functions of these genes (according to Uniprot.org, February 2017) are as follows: *SRC* is a proto-oncogene with roles in embryonic development and cell growth; *DFFB* encodes a DNA fragmentation factor that triggers DNA fragmentation and chromatin condensation during apoptosis; *INHBE* encodes a member of the TGF-beta family and has been shown to inhibit proliferation, and growth of the pancreas and liver; and *HEPACAM3* encodes a protein required for centrosome maturation during cell division. UT - untreated *p<0.05; **p<0.005; ***p<0.0005. Data is represented relative to untreated cells. Data and significance (assessed by one-way ANOVA) is represented relative to UT cells.

**Supplementary Figure S4. Western blot analysis of expression of c-myc in NET cells**. C-myc expression could be detected in BON-1 and H727 cells, but was absent in H720 cells. α-tubulin was used as a loading control.

**Supplementary Figure S5.** **Expression of menin in PNETs of *Men1^L/L^/RIP2-Cre* mice**. Loss of menin, defined as lack of brown staining in the nucleus (red arrows), was observed in PNETs from male and female mice from all treatment groups, JQ1 compound, or vehicle only or JQ1- controls. However, nuclear expression of menin in normal islets of control *Men1^L/L^* mice was observed (black arrows). Images are shown at x400 magnification.

**Supplementary Table S1.**

| **Compound** | **Target** | **Epigenetic function of target** | **IC90 range*** | **Concentration^§^** |
| --- | --- | --- | --- | --- |
| **(-)-JQ1** | Control compound, no target | NA | NA | 20 nM; 50nM; 100nM; 500nM;  1 μM |
| **(+)-JQ1** | Both bromodomains of the BET protein family (BRD2, BRD3, BRD4 and BRDT) | Acetyl lysine binder | 0.5-1 μM | 20 nM; 50nM; 100nM; 500nM;  1 μM |
| **PFI-1** | Both bromodomains of the BET protein family (BRD2, BRD3, BRD4 and BRDT) | Acetyl lysine binder | ND | 20 nM; 50nM; 100nM; 500nM;  1 μM |
| **RVX-280** | Second bromodomain of the BET protein family (BRD2, BRD3, BRD4 and BRDT) | Acetyl lysine binder | ND | 1 μM |
| **C464** | CBP/P300 | Histone acetyltransferase | ND | 1 μM |
| **UNC0638** | Ga9/GLP | Histone methyltransferase | 0.5-1 μM | 1 μM |
| **UNC0642** | Ga9/GLP (improved PK properties to UNC0638) | Histone methyltransferase | 0.5-1 μM | 1 μM |
| **SGC0946** | DOT1L | Histone methyltransferase | 0.1-1 μM | 1 μM |
| **IOX-1** | 2-oxoglutarate oxygenases | Lysine demethylation | 50-100 μM | 100 μM |
| **UNC1215** | L3MBTL3 | Methyl lysine binder | 0.1-1 μM | 1 μM |

*Based on in vitro studies performed by the manufacturer; ^§^Concentration used in this study; SGC – Structural Genomics Consortium; PK – pharmacokinetic; NA- not applicable; ND- not determined

**Supplementary Table S2.**

| **Ingenuity Canonical Pathways** | **-log**  **(p-value)** | **Ratio** | **z-score** | **Molecules** |
| --- | --- | --- | --- | --- |
| 3-phosphoinositide Degradation | 2.68E+00 | 5.76E-02 | NaN | INPP5F,PPP1R14B,PPP1R13B,PPP2R3A,INPP4A,SIRPA,PPFIA4,MTM1 |
| Atherosclerosis Signaling | 2.42E+00 | 5.79E-02 | NaN | CLU,CSF1,PCYOX1,PLB1,ICAM1,MMP1,CXCL8 |
| Role of Macrophages, Fibroblasts and Endothelial Cells in Rheumatoid Arthritis | 2.10E+00 | 3.83E-02 | NaN | SRC,FN1,NFATC2,CSF1,TLR6,WNT4,ICAM1,MMP1,FZD4,CXCL8,PLCG2 |
| Dopamine Receptor Signaling | 2.06E+00 | 6.49E-02 | NaN | PRKAR2B,PPP1R14B,DDC,PPM1L,PPP2R3A |
| Production of Nitric Oxide and Reactive Oxygen Species in Macrophages | 2.02E+00 | 4.47E-02 | 0.707 | PPP1R14B,CLU,PPM1L,PCYOX1,PPP2R3A,SIRPA,PLCG2,FNBP1 |
| Sperm Motility | 1.95E+00 | 5.26E-02 | 0.000 | PRKAR2B,PRKG2,ITPR1,PLB1,PTK7,PLCG2 |
| Regulation of Cellular Mechanics by Calpain Protease | 1.90E+00 | 7.27E-02 | NaN | SRC,CAPN5,CAPN8,TLN2 |
| FAK Signaling | 1.87E+00 | 5.81E-02 | NaN | SRC,CAPN5,CAPN8,TLN2,PLCG2 |
| Integrin Signaling | 1.83E+00 | 4.12E-02 | 0.816 | SRC,CAPN5,TSPAN1,NEDD9,CAPN8,TLN2,PLCG2,FNBP1 |
| Dopamine-DARPP32 Feedback in cAMP Signaling | 1.82E+00 | 4.46E-02 | 0.447 | PRKAR2B,PPP1R14B,PPM1L,PRKG2,ITPR1,PPP2R3A,PLCG2 |
| Synaptic Long Term Depression | 1.61E+00 | 4.41E-02 | -1.633 | PPM1L,PRKG2,ITPR1,PPP2R3A,PLB1,PLCG2 |
| GDNF Family Ligand-Receptor Interactions | 1.59E+00 | 5.88E-02 | NaN | RET,IRS1,ITPR1,PLCG2 |
| D-myo-inositol-5-phosphate Metabolism | 1.58E+00 | 4.35E-02 | NaN | PPP1R14B,PPP1R13B,PPP2R3A,SIRPA,PPFIA4,PLCG2 |
| AMPK Signaling***** | 1.57E+00 | 3.95E-02 | -1.134 | SRC,STRADA,PRKAR2B,PPM1L,ACACB,IRS1,PPP2R3A |
| TREM1 Signaling | 1.55E+00 | 5.71E-02 | NaN | TLR6,ICAM1,CXCL8,PLCG2 |
| Hepatic Fibrosis / Hepatic Stellate Cell Activation | 1.52E+00 | 3.87E-02 | NaN | FN1,CSF1,COL17A1,IGFBP5,ICAM1,MMP1,CXCL8 |
| Role of Tissue Factor in Cancer | 1.50E+00 | 4.67E-02 | NaN | SRC,CYR61,CSF1,MMP1,CXCL8 |
| Glioblastoma Multiforme Signaling | 1.49E+00 | 4.14E-02 | 0.000 | SRC,ITPR1,WNT4,FZD4,PLCG2,FNBP1 |
| Superpathway of Inositol Phosphate Compounds | 1.47E+00 | 3.76E-02 | NaN | INPP5F,PPP1R14B,PPP1R13B,PPP2R3A,SIRPA,PPFIA4,PLCG2 |
| ERK/MAPK Signaling | 1.47E+00 | 3.76E-02 | 0.378 | SRC,PRKAR2B,PPP1R14B,PPM1L,PPP2R3A,TLN2,PLCG2 |
| Gap Junction Signaling | 1.42E+00 | 3.97E-02 | NaN | TUBA1A,SRC,PRKAR2B,PRKG2,ITPR1,PLCG2 |
| p70S6K Signaling***** | 1.35E+00 | 4.24E-02 | -1.342 | SRC,PPM1L,IRS1,PPP2R3A,PLCG2 |

***** Signalling pathways associated with mechanistic target of rapamycin (mTOR) and phosphoinositide 3-kinase (PI3K) signalling.

**Supplementary Table S3.**

| **Up-regulated** | | **Down-regulated** | |
| --- | --- | --- | --- |
| **Log FC** | **Gene** | **Log FC** | **Gene** |
| 4.19 | *HIST2H2BE* | -5.73 | *GPR114* |
| 4.01 | *EFR3B* | -5.67 | *DLL3* |
| 3.96 | *HIST1H2BD* | -4.69 | *BCMO1* |
| 3.83 | *HIST2H2AA3* | -4.56 | *ENPP3* |
| 3.82 | *RP11-509E16.1* | -4.41 | *HEPACAM2* |
| 3.73 | *HIST1H2AC* | -4.29 | *PHEX* |
| 3.73 | *C14orf37* | -3.99 | *ENAM* |
| 3.52 | *MIR7-3HG* | -3.89 | *INSM1* |
| 3.50 | *CASC1* | -3.85 | *USP41* |
| 3.49 | *IQUB* | -3.83 | *AP000345.1* |
| 3.48 | *TMEM132B* | -3.72 | *ST18* |
| 3.47 | *MORN5* | -3.72 | *INHBE* |
| 3.26 | *ROPN1L* | -3.41 | *RP11-279F6.1* |
| 3.17 | *KIAA1683* | -3.40 | *ACSL5* |
| 3.15 | *HIST1H2AG* | -3.39 | *XXbac-B33L19.6* |
| 3.11 | *RP11-196G18.22* | -3.39 | *RP11-290F20.1* |
| 3.08 | *HIST2H2BC* | -3.25 | *GPX2* |
| 3.02 | *ZCCHC24* | -3.18 | *LINC01014* |
| 2.99 | *ACSL1* | -3.15 | *GPNMB* |
| 2.90 | *PEG10* | -3.12 | *COL17A1* |

**Supplementary Figure S1**


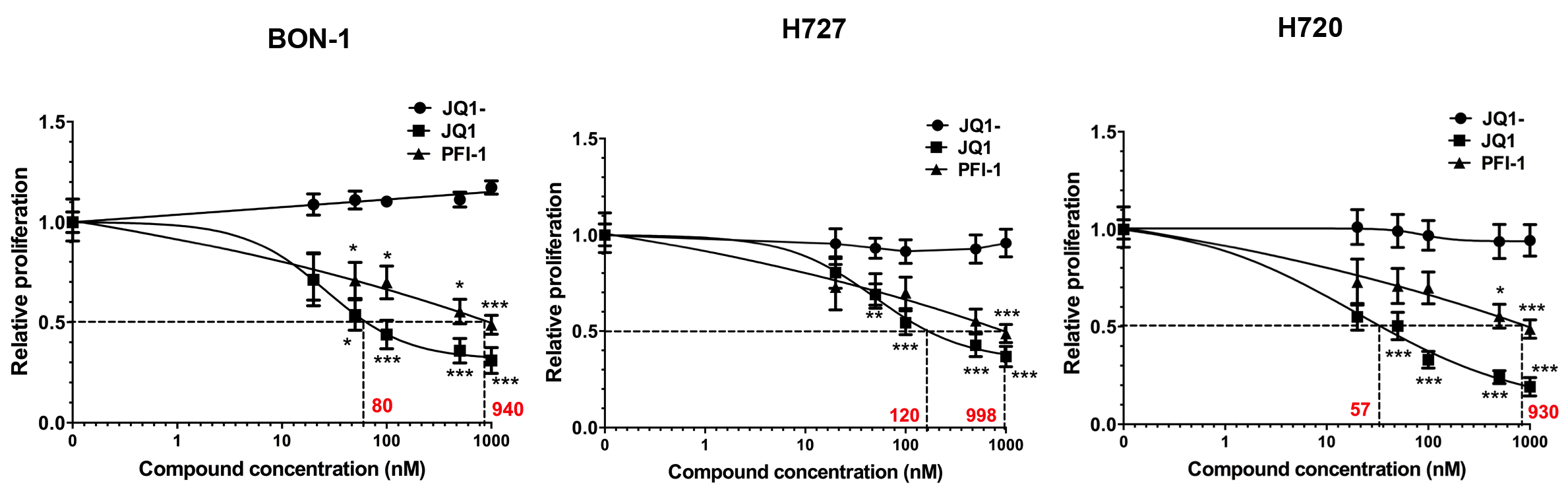


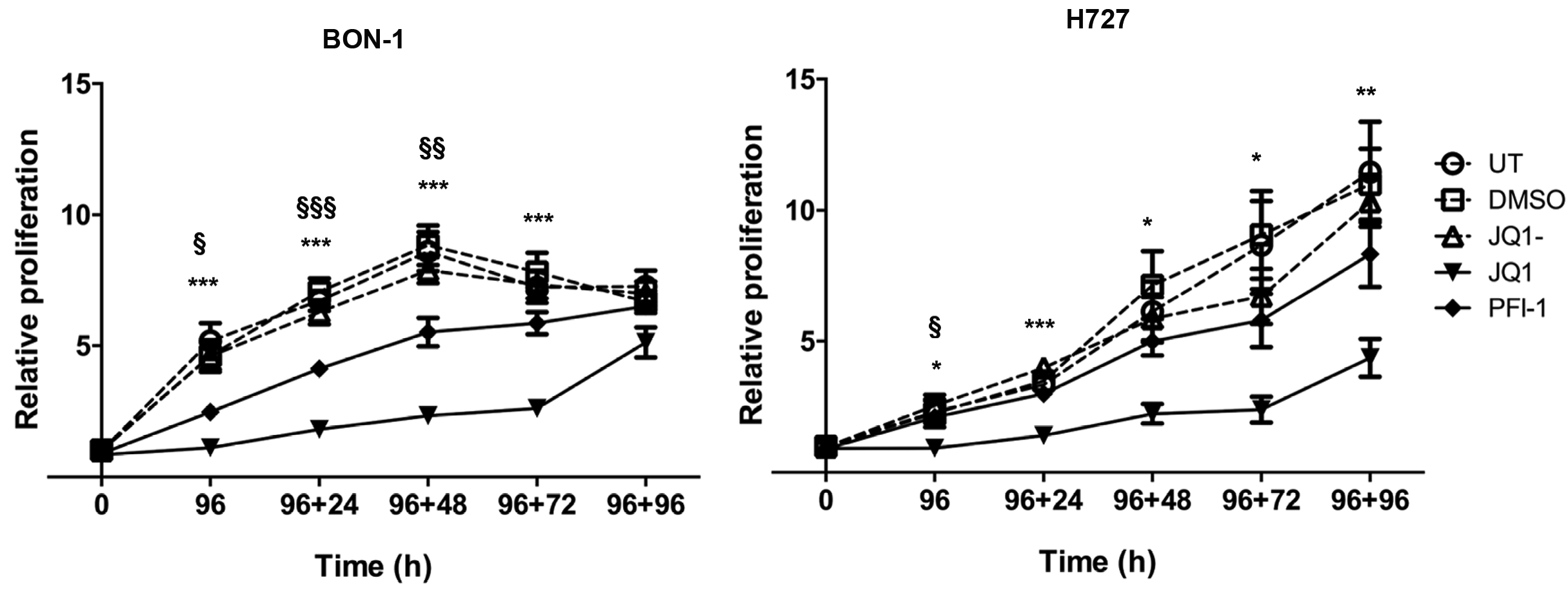


**Supplementary Figure S2**

**Supplementary Figure S3**


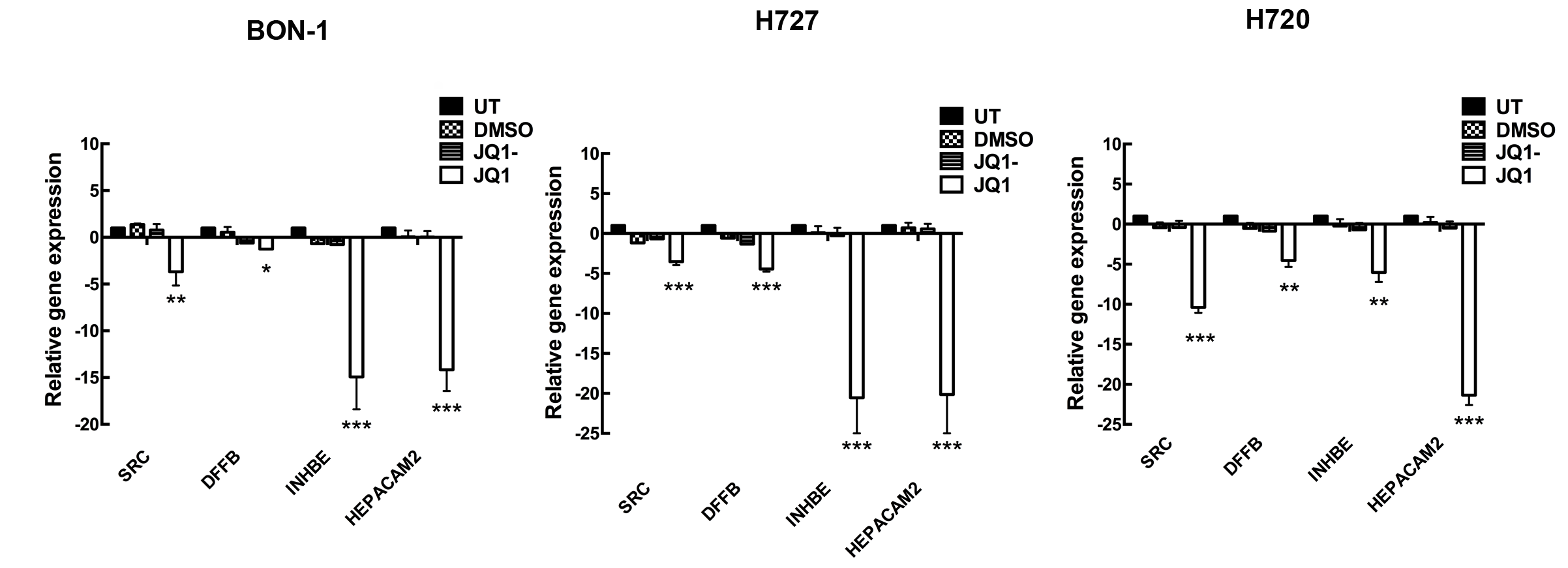


**Supplementary Figure S4**


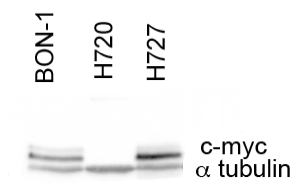


**Supplementary Figure S5.**

**
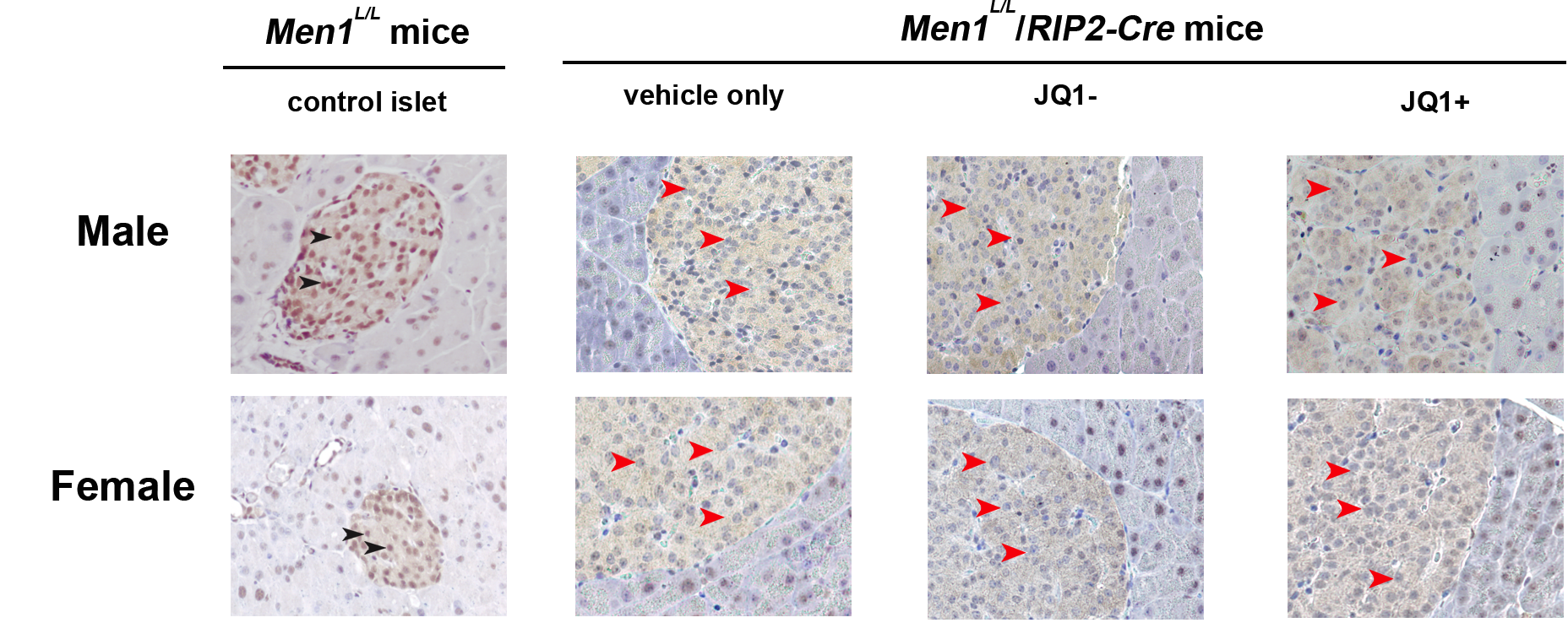
**
